# Wide range and highly linear signal processed systematic humidity sensor array using Methylene Blue and Graphene composite

**DOI:** 10.1038/s41598-021-95977-6

**Published:** 2021-08-17

**Authors:** Muhammad Umair Khan, Gul Hassan, Rayyan Ali Shaukat, Qazi Muhammad Saqib, Mahesh Y. Chougale, Jungmin Kim, Jinho Bae

**Affiliations:** 1 Department of Ocean System Engineering, Jeju National University, 102 Jejudaehakro, Jeju, 63243 Korea; 2 Centre for Advanced Electronics and Photovoltaic Engineering (CAEPE), International Islamic University, H-10, Islamabad, 44000 Pakistan

**Keywords:** Engineering, Materials science, Nanoscience and technology

## Abstract

This paper proposes a signal processed systematic 3 × 3 humidity sensor array with all range and highly linear humidity response based on different particles size composite inks and different interspaces of interdigital electrodes (IDEs). The fabricated sensors are patterned through a commercial inkjet printer and the composite of Methylene Blue and Graphene with three different particle sizes of bulk Graphene Flakes (BGF), Graphene Flakes (GF), and Graphene Quantum Dots (GQD), which are employed as an active layer using spin coating technique on three types of IDEs with different interspaces of 300, 200, and 100 µm. All range linear function (0–100% RH) is achieved by applying the linear combination method of nine sensors in the signal processing field, where weights for linear combination are required, which are estimated by the least square solution. The humidity sensing array shows a fast response time (T_res_) of 0.2 s and recovery time (T_rec_) of 0.4 s. From the results, the proposed humidity sensor array opens a new gateway for a wide range of humidity sensing applications with a linear function.

## Introduction

Relative humidity (RH) is an important parameter to be under observations in the environment for different applications like industry, food, health, and even space applications^1^. The humidity sensor can measure and detect different parameters such as impedance, resistance, and capacitance^2,3^. For humidity sensing, many researchers are working to improve the following parameters like hysteresis, temperature dependence, linearity, sensitivity, reproducibility, time response, and low production cost^3^. It is impossible to fabricate a humidity sensor, that can deliver all the above mentioned parameters. In these expectations, for the commercialization of humidity sensors, several researchers are working on different designs based on interdigital electrodes (IDEs)^4^, capacitance-based design^5^, piezoelectric^6^, and surface acoustic waves^7^. In all of these proposed humidity designs, the IDEs based humidity sensors are more preferred for environment monitoring as it provides higher sensitivity by efficiently detecting any change in active film characteristics on all electrode fingers^3^. IDEs are easy to fabricate and a single sensing layer is required for environment monitoring^8^. In humidity sensor, RH presents the percent ratio of actual vapor density and saturation vapor density, and when air is fully saturated with water vapor and cannot able to hold any more water vapor, this environment condition is called fully saturated with 100% RH^3^.

So far, many researchers are studying different materials to detect RH like 2D materials^9^, metal oxide^10^, biomaterials^1^, organic materials^11^, inorganic materials^2^, organic–inorganic materials^12^, and other nanocomposites materials^3^ with desired properties that can exhibit the target performance parameters when employed as active elements for humidity sensing. This approach helps to achieve a few targets, but at the same time ignores a few parameters like user range of detection, friendly interface, response time, etc.^1,4^. Other researchers are mainly focusing on working mechanism, humidity sensor design, humidity sensor configuration (like series–parallel configuration), while they are paying less attention to material aspect^2,13^. Whereas, few researchers are applying a new type of composite like Graphene/(Zinc oxide)^2^, which is impedance based humidity sensor in parallel configuration. Here, it is showing a good response at middle range of humidity level, but it is not sensitive for a low range of humidity, due to very high impedance at a low range of humidity. In order to increase the sensitivity of the impedance based humidity sensor, generally insulator material Polyvinyl phenol (PVP) is mixed with conductive material Poly(3,4-ethylenedioxythiophene)-poly(styrenesulfonate) (PEDOT:PSS)^14^, in which impedance of PVP decreased by mixing PEDOT:PSS. However, the sensitivity of the humidity sensor is still not sufficient for the full range. To improve the sensitivity of the humidity sensor at a low range, two interdigital electrodes are used in series^13^, in which one humidity sensor is coated with PEDOT:PSS and the other coated with MoS_2_ with RH percent (%) range from 0 to 80% with almost linear response. However, the sensor needs several improvements for a full range of humidity detection from 0 to 100% RH with linear response.

**Figure 1:**
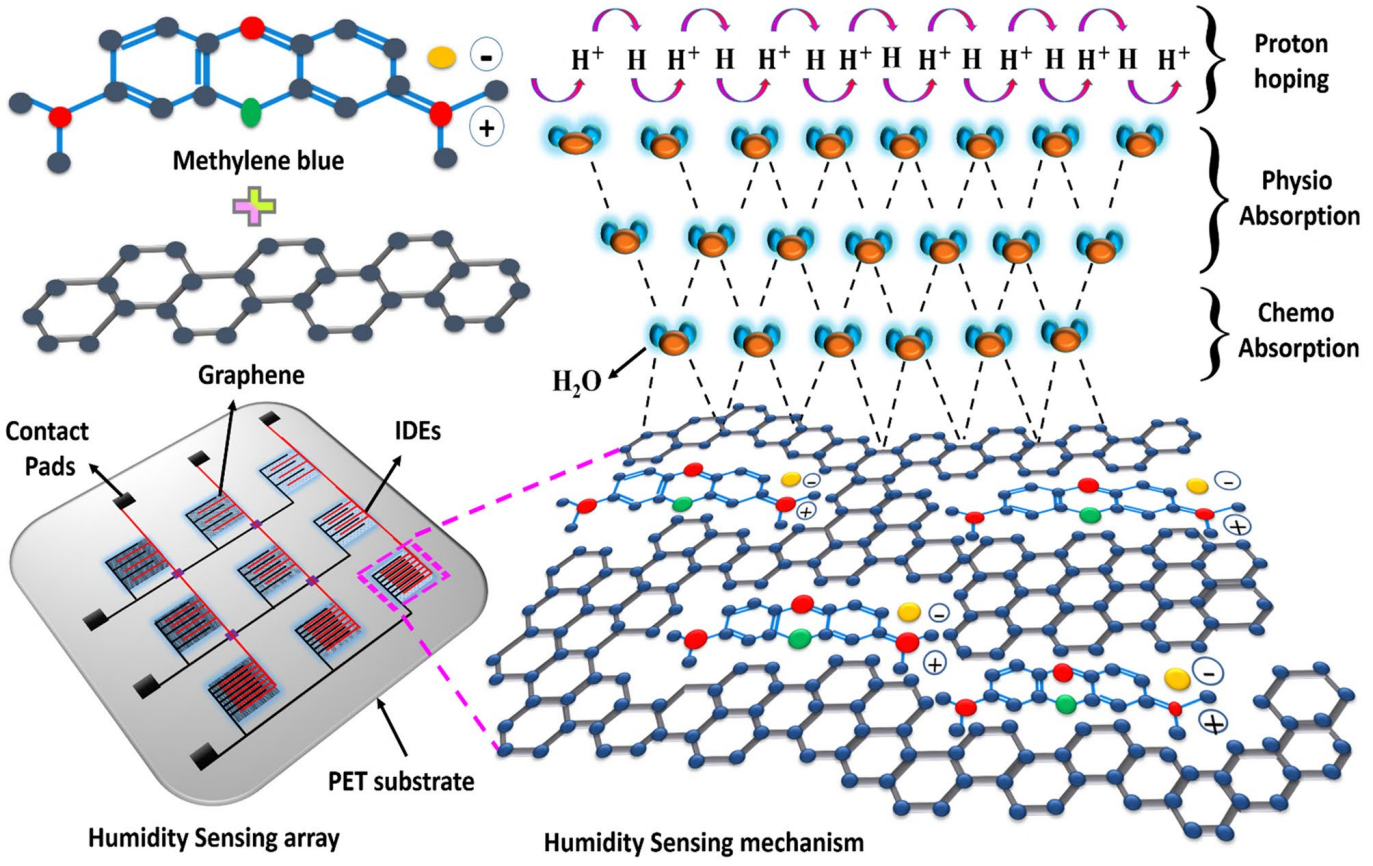
Humidity sensing array and sensing mechanism.

To cover the chemical and physical aspect of all ranges of RH detection, composite inks of Methylene Blue (MB) with three different particle sizes of Graphene are utilized, and different interspaces of three types of IDEs. In this paper, a 3 × 3 sensing array is demonstrated as shown in Fig. 1. Graphene is a highly conductive material due to the p-orbital electrons, which results in high carrier mobility with nearby atoms^3^. Graphene is widely used in various sensor applications, and it is very sensitive to immediate environment change, due to the presence of pi-bond^15–17^. However, Graphene is not sufficiently sensitive to  a wide range of RH, because of its high conductivity^3^. To improve this problem, MB and Graphene nanocomposites are applied, which increases the sensitivity and sensing range as shown in Fig. 1. Here, MB has very good absorptivity towards environment change and is widely used as the sensing material for chemical sensors due to the high molar absorption coefficient^18^. The MB and Graphene composite with different particle sizes is utilized in the ratio of 1:1 as following MB and bulk Graphene flakes composite (MB/BGF), MB and Graphene flakes composite (MB/GF), MB and Graphene quantum dots composite (MB/GQD). To design all range systematic humidity sensing array, inkjet printing, and spin coating technique is utilized. The fabricated 3 × 3 sensing array is tested with a homemade humidity sensing setup. The overall combined impedance response of IDEs physical connection in parallel of all columns and rows provides a wide piecewise linear, and a perfect linear function can be obtained,  and further all array IDEs are processed using the linear combination method in the signal processing field to achieve all range linear response. The proposed sensing array shows fast response time (T_res_) and recovery time (T_rec_). These results proposes the design and fabrication method of a novel humidity array sensor with linear characteristics on all sensing range.

## Materials and methods

### Materials preparation

The material for humidity sensing is one of the main parameters to analyze the change in impedance with the change in water vapors blown on it and this change is easily detected with fingers of IDEs. Three inks for the active layer were prepared as follows; composite of MB/BGF, MB/GF, and MB/GQD was synthesized as shown in Supplementary Fig. S1a. The graphite powder, Methylene Blue (MB) powder, Graphene quantum dots (GQD) ink, Acetonitrile, and N-methyl-pyrrolidone (NMP) were purchased from Sigma Aldrich, South Korea. To fabricate the sensing array, a Polyethylene terephthalate (PET) substrate was purchased from AgIC paper Electronics. The 3 × 3 IDEs for sensor array structure was patterned on PET substrate using 50% dispersion of Ag nanoparticle ink within triethylene glycol monomethyl ether (TGME). The 0.05 g of Graphene powder was dispersed in 10 ml of NMP. The solution was probe sonicated for 10 min with 1 s on the pulse and 2 s off pulse with 20 kHz of probe frequency to break large Graphene particles into small particles and then ink was magnetically stirred for 4 h and bath sonicated for 40 min at room temperature. This ink contains Graphene flakes in bulk form, that is why this ink is named Bulk Graphene Flakes (BGF). The ink was further purified with centrifugation at 5000 rpm for 30 min and the supernatant was separated from sediment. This ink contains Graphene flakes (GF) in a very small amount. The 1 mg/ml dispersion Graphene quantum dots (GQD) in water was purchased from Sigma Aldrich. The 1 mM MB ink was prepared by mixing in acetonitrile and bath sonicated for 30 min at 50 °C and magnet stirred for 60 min. The chemical structure of MB and Graphene and prepared ink are shown in Fig. 1. The Graphene and Methylene Blue are mixed in 1:1 to form 3 types of inks following MB/BGF, MB/GF, and MB/GF. All three inks are placed on a magnetic stirrer for 60 min to obtain a uniform dispersion of MB with BGF, GF, and GQD as shown in Supplementary Fig. S1a.

### Interdigital electrode fabrication

In IDEs on the proposed sensor array, they contain many electrode figures, which improves the sensitivity. IDEs are more sensitive as compared to two electrodes because all figures electrodes are in parallel to sense the change in the electric property of the active layer. To fabricate the 3 × 3 humidity sensing array on PET substrate, the IDEs were designed using EAGLE version 9 in Dxf format with the same electrode spacing in each row and different electrode spacing in each column like 300, 200, and 100 μm. The 3 × 3 IDEs sensing array design file is converted to a BMP file and Dimatix Drop Manager converts the BMP file into an open file format. The ptn file was uploaded to the inkjet printing system. The 1 pL 16 nozzles cartridge was filled with 3 ml of Ag ink. The 25 V was applied on cartridge nozzles for stable printing. The temperature of the printing platform was set at 40 °C as shown in Supplementary Fig. S1b. The proposed 3 × 3 IDEs sensing array was fabricated on PET substrate using an inkjet printing system and cured at 60 °C for 2 h. The length and width of each IDE in a sensing array are 12 mm and 20 mm, respectively. The length and width of each figure are 10 mm and 100 μm, respectively. In the 3 × 3 sensor array, the finger spacing of IDEs in row 1, row 2, and row 3 is as following 300, 200, and 100 μm, respectively, as shown in Supplementary Fig. S1d, and the fabricated sensor array is shown in Supplementary Fig. S1e.

### Spin coating of active layer

The nanocomposites of MB/BGF, MB/GF, and MB/GQD are used to fabricate the active layer on the top silver patterned IDEs by using spin coating technology as shown in Supplementary Fig. S1c. The ink 1 based on MB/BGF was spin-coated on each IDEs of column 1 at 2500 rpm, which contains the following sensors HR1C1, HR2C1, and HR3C1. The ink 2 composite consists of MB/GF was spin-coated on each sensor of column 2 at 25,000 rpm, which contains the following sensors HR1C2, HR2C2, and HR3C2. The ink 3 MB/GQD composite was spin-coated on each sensor of column 3 at 2500 rpm on the following sensors HR1C3, HR2C3, and HR3C3 shown in Supplementary Fig. S1c. The active layer was cured at 100 °C for 30 min.

### Characterizations

The surface morphology of Ag, MB, BGF, GF, and GQD were characterized with TESCAN MIRA 3 scanning transmission electron microscope (STEM). The element composition of Ag, MB, and BGF, GF, and GQD were analyzed with energy-dispersive X-ray spectroscopy (EDS). The 2D and 3D nano-profile of IDEs and composite films of MB/BGF, MB/GF, and MB/GQD were analyzed with NV-2000 Universal non-contact surface profiler. The structural and chemical investigation of MB and Graphene were characterized by using a Lab Ram HR Evolution Raman spectrometer (Horiba Jobin-Yvon, France). The impedance response of the sensing array towards change in relative humidity was recorded in a homemade environmental controlled chamber.

## Results and discussion

The schematic of the active layer mechanism based on IDEs is explained in Fig. 1 and the equivalent model of 3 × 3 humidity sensing array is discussed in Supplementary Fig. S2a. The mechanism based on the chemical properties of the composite film is explained in Fig. 1. The terminals of the IDEs are connected with an LCR meter, which reads the impedance of the sensor with a variation of % RH. The phenomena of the Graphene and Methylene Blue composite film are shown in Fig. 1. During humidification active layer adsorb more water vapors and decreases the impedance due to the physio absorption phenomena of water vapors with Methylene Blue and Graphene as shown in Fig. 1. Likewise, when water vapors are desorbed from the active layer during dehumidification as the result impedance of the sensor increases. The chemisorbed water molecule layer is formed on the sensing layer (based on Methylene Blue and Graphene composite), results in the formation of OH^–^ ions at the low level of humidity, and acts as an electron donor. During continuous absorption of water molecules, the physio absorbing layer is formed on the chemisorbed water molecule layer as shown in Fig. 1. The proton hoping mechanism occurs on the physiosorbed layer as given in Eq. (1).

1 $${\text{H}}^{ + } + {\text{H}}_{2} {\text{O}} \to {\text{H}}_{2} {\text{O}} + {\text{H}}^{ + }$$

Under the presence of condensed water molecules on the active layer leads to the formation of a second physiosorbed layer, which helps to achieve proton hopping. Moreover, a water molecule can dissociate into hydronium OH^−^ ions at the high level of humidity as given reaction given in Eq. (2).

2 $${\text{H}}_{2} {\text{O}} + {\text{H}}_{2} {\text{O}} \to {\text{H}}_{3} {\text{O}}^{ + } + {\text{OH}}^{ - }$$

In steady-state, the electrical resistance of a few Graphene flakes is very high. However, the resistance of the Graphene film can be decreased, if flakes are electrically connected in the presence of water vapors^2,19^. Hence, to make the electrical connection between Graphene flakes to flakes MB is used, which results in blinds the flakes together. Therefore, in the absence of water vapor composite of MB with Graphene has a very high impedance. When water vapors are blown on the surface of composite film, MB absorbs the water vapors and decreases the impedance, which results in a connection between Graphene flakes^3^ as shown in Fig. 1. The sensitivity of Graphene is mainly due to the presence of electrostatic forces with water vapors that can create a dipole. On the other hand, MB also has a humidity sensing property^20^. The composite of MB with Graphene provides two advantages, first, MB absorbs water vapors and decreases the overall film impedance. Secondly, MB fills the gap between Graphene flakes and completes the current path between Graphene flakes. The Impedance of the composite sensing layer based on MB/BGF or MB/GF or MB/GQD collected through IDEs to enhance the sensitivity as shown in Fig. 1. The electric field simulation of the sensing array is presented in Supplementary Fig. S2b–e.

**Figure 2:**
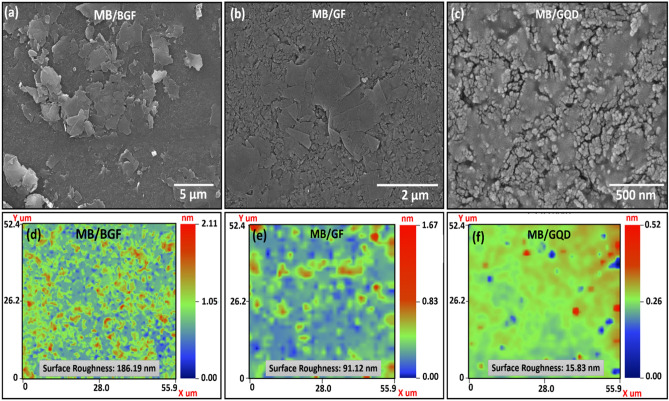
The Surface morphology of composite film, (**a**) MB/BGF at 5 μm showing Graphene flakes in bulk form in a composite film, (**b**) MB/GF composite film at 2 μm, and (**c**) MB/GQD film at a magnification of 500 nm, respectively. The 2D Nano profile of composite films, (**d**) MB/BGF, (**e**) MB/GF, and (**f**) MB/GQD to confirm the surface roughness.

The composite film of MB/BGF is observed at a magnification level of 5 um a shown in Fig. 2a, which clearly shows the BGF in a composite film. Due to the presence of bulk flakes composite film has maximum film roughness. The MB/GF film is observed at 2 μm as shown in Fig. 2b, the flakes in a composite film, which clearly shows that flakes are properly merged in a composite film, which results in a decrease of film roughness. The composite film of MB/GQD is shown in Fig. 2c at a magnification level of 500 nm and the composite film is uniform. The 2D nano-profile is performed to observe the surface roughness of each composite like MB/BGF, MB/GF, and MB/GQD. The surface roughness of MB/BGF is Ra ~ 186.19 nm as shown in Fig. 2d, the surface roughness of MB/GF is Ra ~ 91.12 nm as shown in Fig. 2e and the surface roughness of MB/GQD is 15.83 nm as shown in Fig. 2f. These results clearly show that, with a decrease in particle size of Graphene, we have a decrease in surface roughness in a composite film. The surface morphology, EDS, and chemical characterization of Ag, MB, BGF, GF, and GQD are discussed in Supplementary Fig. S3, S4, and S5.

**Figure 3:**
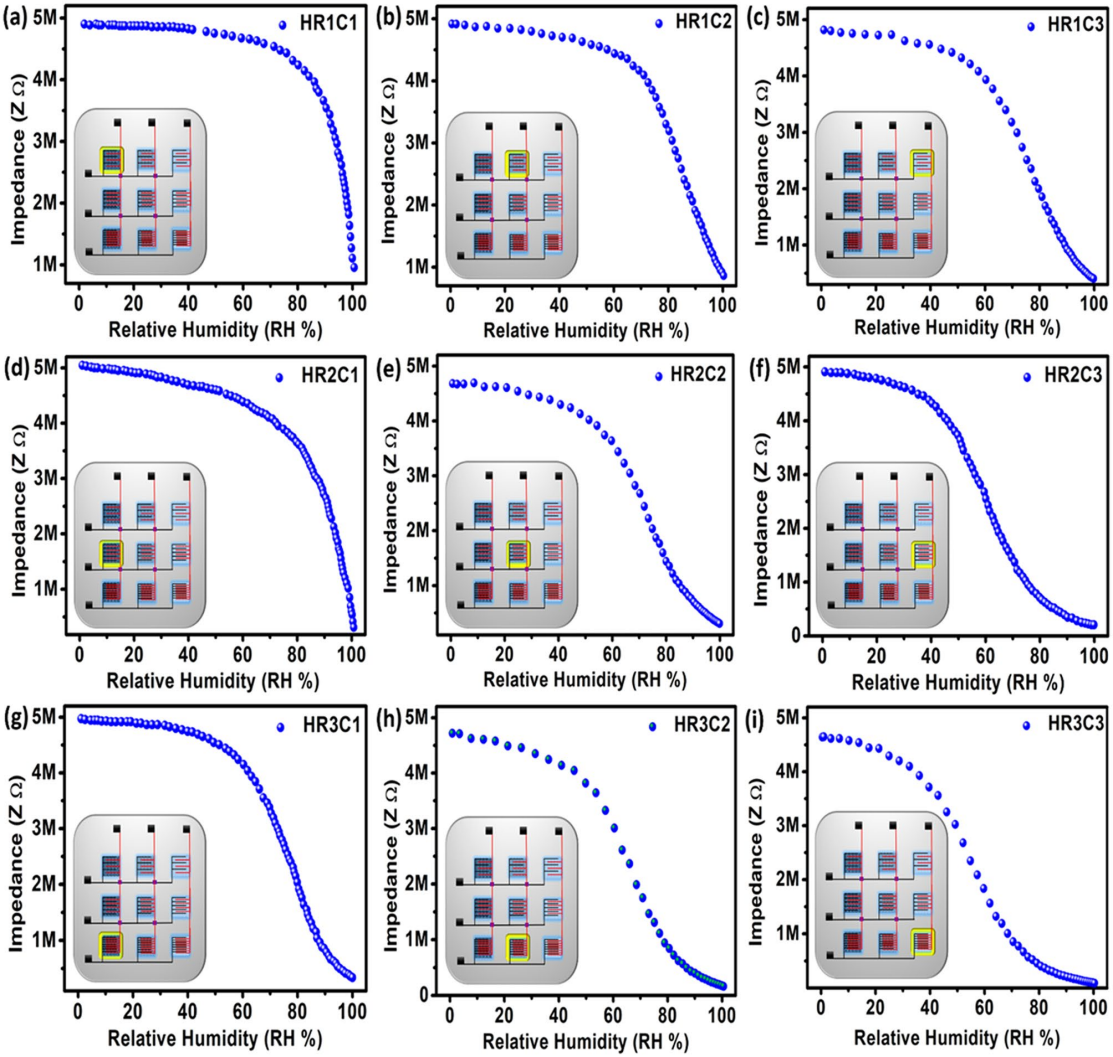
The 3 × 3 humidity sensing array showing sensors as following row 1 (**a**) HR1C1, (**b**) HR1C2, and (**c**) HR1C3 with 300 μm spacing. The row 2 contain following sensors (**d**) HR2C1, (**e**) HR2C2, and (**f**) HR2C3 with 200 μm spacing. The row 3 contain following sensors (**g**) HR3C1, (**h**) HR3C2, and (**i**) HR3C3 with 100 μm spacing.

The humidity sensing setup is discussed in Supplementary Fig. S6. The impedance response of the 3 × 3 humidity sensor array was analyzed at 1 kHz from 0 to 100% RH as shown in Fig. 3. These results clearly show that each IDEs in a sensing array changes its impedance level with an increase in humidity level. The impedance state of the IDEs is inversely proportional to the test frequency as given by (1), where Z, *f*, C, and R represent impedance, test frequency, capacitance, and resistance of the IDEs, respectively, and $\mathrm{j}=\sqrt{-1}$ as given in Eq. (3).

3 $$Z = R + \frac{1}{j2\pi fC}$$

Here, the usefulness of this mathematical model is proved in Figs. S7 and S8 of the supplementary information. The different particle sizes of Graphene composite with MB quite improved the sensing response on different electrode spacing, however, all range line humidity sensing response from 0 to 100% RH is a quite big challenge and researchers are still working to improve the sensing response of humidity sensor. In this work, we adopted three approaches to archiving linear humidity response: (i) 3 × 3 humidity sensing array is designed with different electrode spacing, (ii) different particles size of Graphene is used with MB to achieve linear impedance response, and (iii) to achieve wide piecewise range linear humidity sensor, the sensors are connected in different combination (of each column, and each row) in parallel.

In the proposed sensor array, column 1 as shown in Fig. 3 coated with BGF/MB, with following sensors HR1C1 (300 μm), HR2C1 (200 μm spacing) and HR3C1 (100 μm spacing) sensors with different electrode spacing.  The sensor HR1C1 impedance response is linear from 80% RH to 100% as shown in Fig. 3a, sensor HR2C1 impedance response is linear from 70% RH to 100% as shown in Fig. 3d and sensor HR3C1 impedance response is linear from 50% RH to 100% as shown in Fig. 3g. The composite ink based on MB/GF is further processed and the bulk form of Graphene flakes is removed with centrifugation and probe sonication. The column 2 coated with GF/MB using following sensors HR1C2 (300 μm), HR2C2 (200 μm) and HR3C2 (300 μm) has improved the detection margin at a lower range of humidity from 0 to 95% RH, and it saturates from 95 to 100% RH as shown in Fig. 3. The sensor HR1C2 impedance response is linear from 70 to 100% RH as shown in Fig. 3b, sensor HR2C2 is linear impedance response from 40 to 100% RH as shown in Fig. 3e and the sensor HR3C2 has a linear impedance response from 20 to 100% RH as shown in Fig. 3h. The column 3 coated with GQD/MB in parallel using the following sensors HR1C3 (300 μm), HR2C3 (200 μm) and HR3C3 (100 μm), the overall sensitivity of the sensor improved at the lower range of RH and response is highly piecewise linear as shown in Fig. 3. The Sensor HR1C3 impedance response is linear from 50 to 100% RH as shown in Fig. 3c, sensor HR2C3 impedance response is linear from 30 to 100% RH as shown in Fig. 3f and the sensor HR3C3 impedance response is linear from 5 to 100% RH as shown in Fig. 3i.

**Figure 4:**
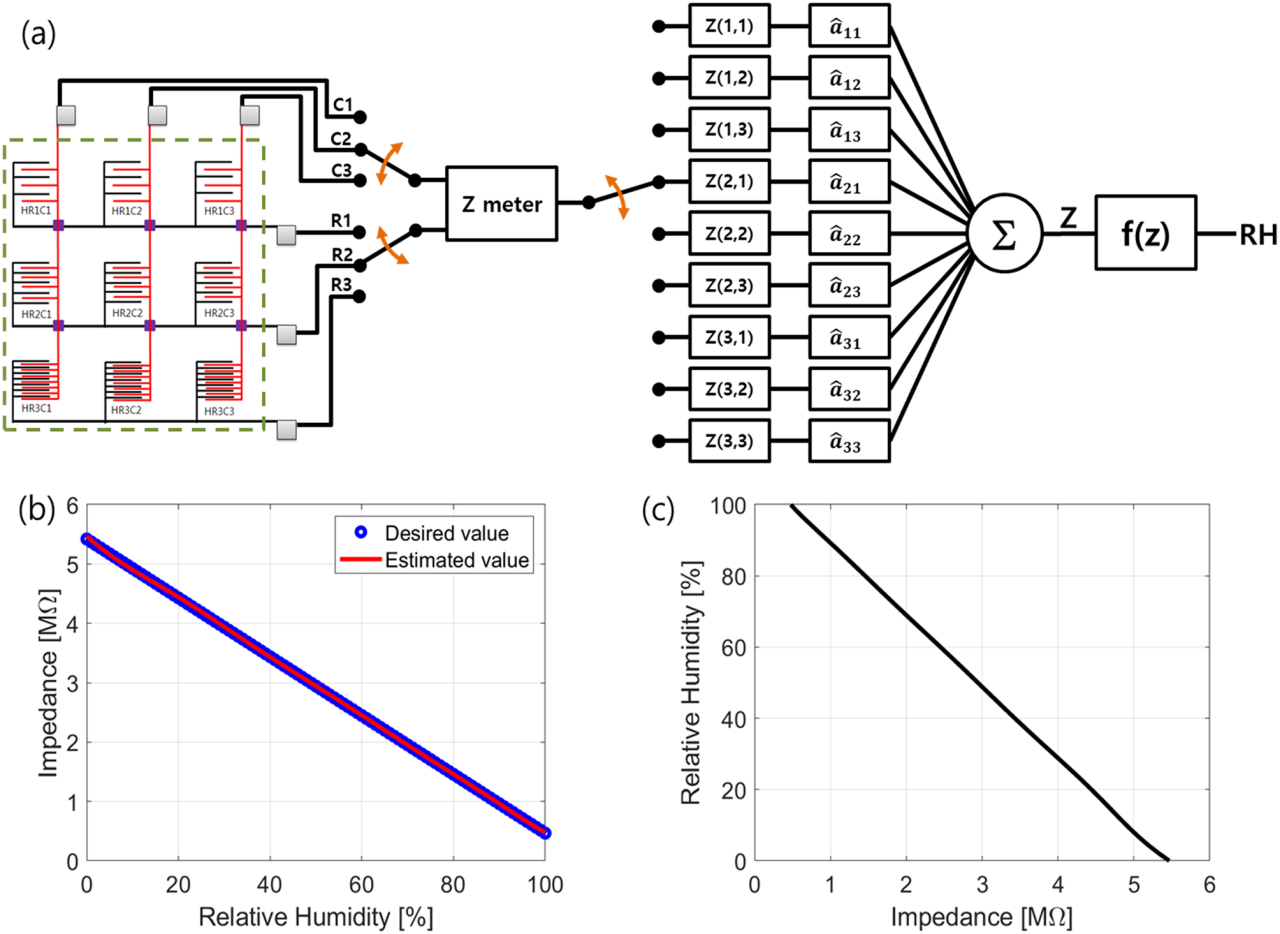
(**a**) Schematic diagram to find an RH value from the measured data of the proposed sensor array. (**b**) For all range linear function, comparison the ideal linear curve and the plotted estimated function by using the calculated estimate weight values $(\underline{{\hat{a}}} )$ in Figure S8 of the supplementary information. (**c**) f(z) to find relative humidity.

The single sensor response in the sensing array lags in linearity and full range sensing response. To solve this problem after sensor and material design, we have adopted the wide range systematic humidity sensing array processing technique to solve sensor linearity in all ranges. Hence, the paper proposes the linear combination method in the signal processing field of the nine sensor elements to get all range linear functions as shown in Fig. 4a. Here, impedance (Z) meter read values from all sensors (where we assume that switching time to read them are sufficiently fast because these are detected before humidity is changed.), and these measured impedances are memorized in registers ${\text{Z}}({\text{i}},\;{\text{j}})$, was, i = {1, 2, 3} and j = {1, 2, 3}. The estimated weights, $\hat{a}_{ij} = \left\{ {\hat{a}_{11} ,\; \hat{a}_{12} , \;\hat{a}_{13} , \;\hat{a}_{21} , \;\hat{a}_{22} , \;\hat{a}_{23} , \;\hat{a}_{31} , \;\hat{a}_{32} ,\; \hat{a}_{33} } \right\}$ = {− 1.395919139453048, 1.509000728240909, 12.98669965832261, 0.521398705626367, 1.16948647255811, − 2.648766910355043, − 12.53225673113707, − 0.002212571475620711, 2.067765966401583e} are calculated by applying the least square solution^21^ as described in Fig. S8 of the supplementary information. Figure 4b shows the estimated red line is exactly matched with the ideal linear curve (blue circles). Using these memorized values and estimated weights, we can find an impedance, $Z = \hat{a}_{11} Z(1,\;1) + \hat{a}_{12} Z(1,\;2) + \cdots + \hat{a}_{33} Z(3,\;3)$, and RH can calculate through f(z) with a function as shown in Fig. 4c. For example, when the impedance values are used at about 49.5% and 61.5%, there are detected as the calculated RHs are 49.56% and 61.37% by the proposed sensor array. We are sure that these results can help to apply a sensor array processing technique to improve its performance like a wide range of humidity sensing applications with a linear function.

**Figure 5:**
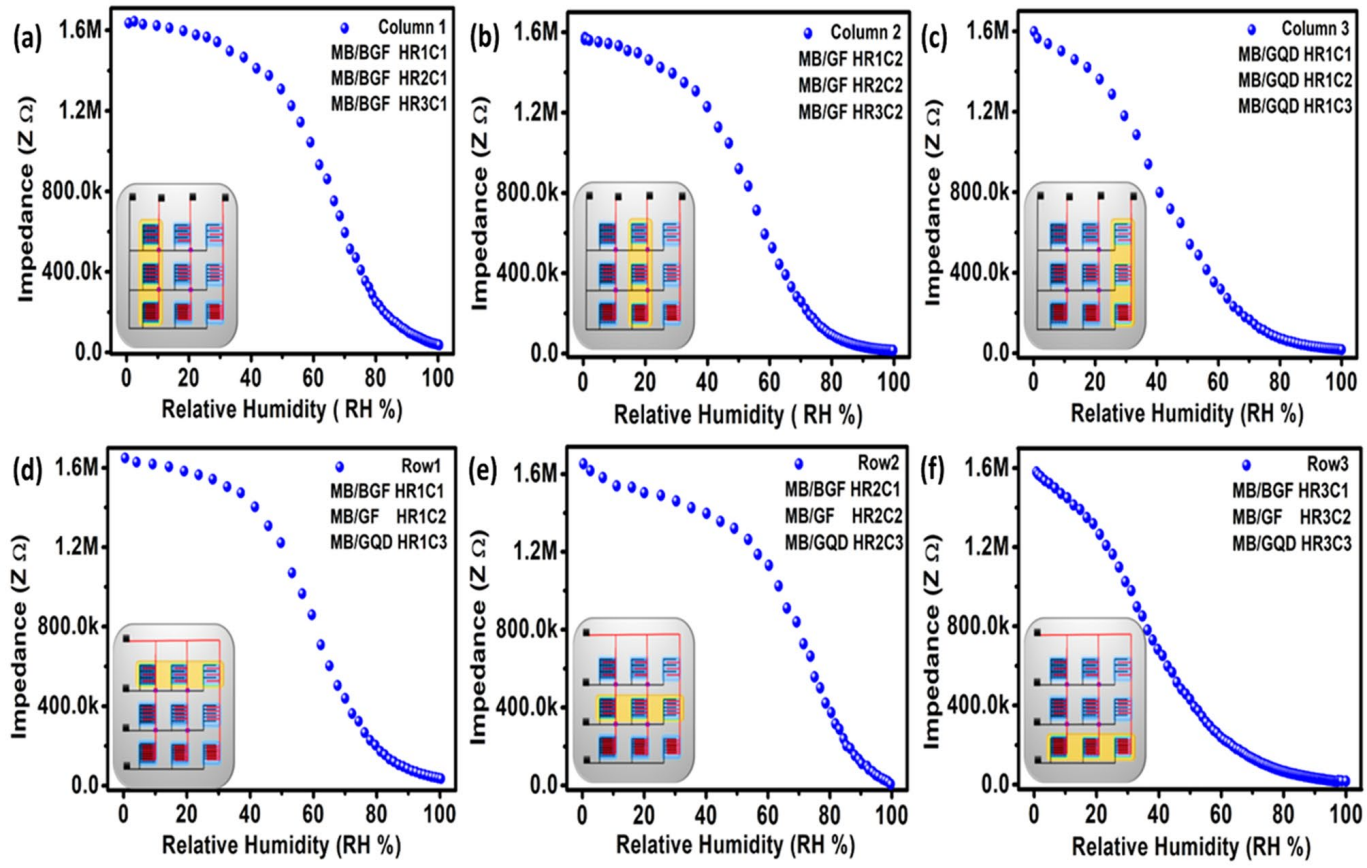
Impedance response of parallel combination of (**a**) column 1, (**b**) column 2 and (**c**) column 3, (**d**) row 1, (**e**) row 2, and (**f**) row 3.

The wider piecewise linear function can also be obtained by the parallel combination of the humidity sensor elements as shown in Fig. 5. In the proposed sensor array, column 1 coated with BGF/MB, with the parallel combination of the following sensors HR1C1 (300 μm), HR2C1 (200 μm spacing), and HR3C1 (100 μm spacing) sensors with different electrode spacing, the overall response becomes more sensitive and wide piecewise linear function as shown in Fig. 5a for the full range from 0 to 100% RH. The combined effect of column 2 coated with GF/MB, in parallel using the following sensors HR1C2 (300 μm), HR2C2 (200 μm) and HR3C3 (300 μm) has improved the detection margin at a lower range of humidity from 0 to 95% RH, and it saturates from 95 to 100% RH as shown in Fig. 5b. The combined effect of column 3 coated with GQD/MB in parallel using the following sensors HR1C3 (300 μm), HR2C3 (200 μm) and HR3C3 (100 μm), the overall sensitivity of the sensor improved at a lower range of RH and response is highly piecewise linear from 0 to 95% RH and it saturates from 90 to 100% RH as shown in Fig. 5c. The combination effect of row 1 with 300 μm spacing in parallel combination using the following sensors HR1C1 (coated with MB/BGF), HR1C2 (coated with MB/GF), and HR1C3 (Coated with MB/GQD), has improved the wide piecewise linear range in overall impedance response 0–100% RH as shown in Fig. 5d. The combined effect of row 2 with 200 μm spacing in parallel combination using following sensors HR2C1 (coated with MB/BGF), HR2C2 (coated with MB/GF), and HR2C3 (coated with MB/GQD), the overall sensitivity and response become more linear from 0 to 100% RH as shown in Fig. 5e. The combined effect of row 3 with 100 μm spacing in parallel combination using following sensors HR3C1 (coated with MB/BGF), HR3C2 (coated with MB/GF), and HR3C3 (coated with MB/GQD), the overall response is highly piecewise linear from 0 to 90% RH as shown in Fig. 5f.

**Figure 6:**
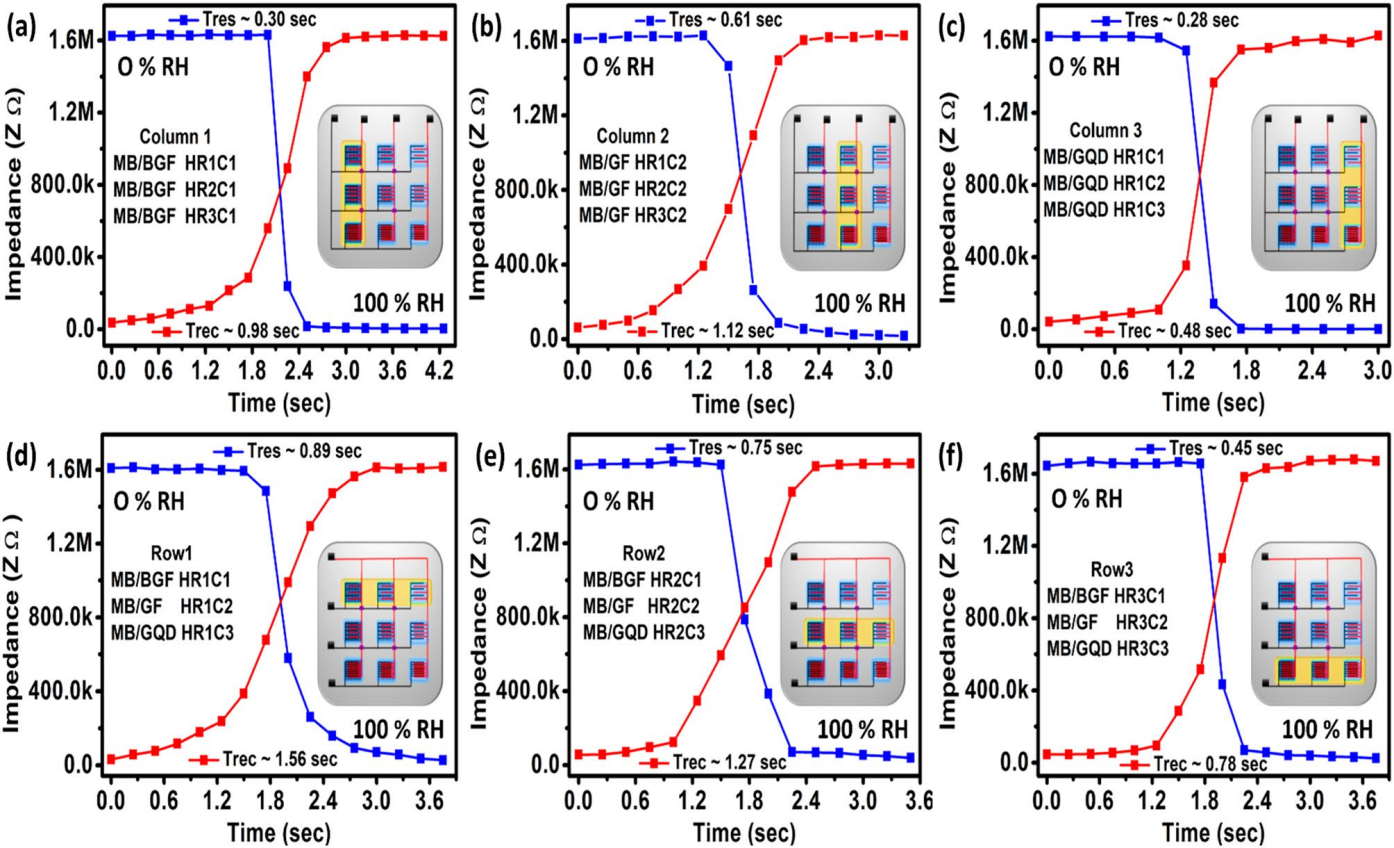
Transient response of (**a**) column 1, (**b**) column 2, (**c**) column 3, (**d**) row 1, (**e**) row 2, and (**f**) row 3.

The transient response of 3 × 3 humidity sensing array is shown in Fig. 6, in which humidity level increased from 0 to 100% RH with humidifier and decreased from 100 to 0% RH with N_2_ gas and response and recovery time of each sensor is recorded^22^. Transient response ensures that how much sensor is sensitive to the sudden change in % RH. The T_res_ and T_rec_ of column 1 are 0.30 s and 0.98 s, respectively as shown in Fig. 6a. The T_res_ and T_rec_ of column 2 are 0.61 s and 1.12 s, respectively as shown in Fig. 6b. The T_res_ and T_rec_ of column 3 are 0.28 s and 0.48 s, respectively as shown in Fig. 6c. The T_res_ and T_rec_ of row 1 are 0.89 s and 1.56 s, respectively as shown in Fig. 6d. The T_res_ and T_rec_ of row 2 are 0.75 s and 1.27 s, respectively as shown in Fig. 6e. The T_res_ and T_rec_ of row 3 are 0.45 s and 0.78 s, respectively as shown in Fig. 6f.

In Table 1, we have discussed a detailed comparison of array-based sensors. The parallel sensor array based on Graphene and ZnO composite operates in a range between 0–85% RH^2^. The impedance response is highly nonlinear for a low range of RH with slow response and recovery time ~ 1 s and ~ 2 s, respectively. The Graphene and Methyl Red composite-based humidity sensor improved the detection range from 5–95% RH, with fast response and recovery time of 0.25 s and 31 s, respectively^3^. Further improvement was made using two sensors in series by using 2D material MoS_2_ and organic conductive polymer PEDOT:PSS^13^. The series combination significantly improved the sensor linearity in a range of 0–80% RH with response time ~ 0.5 s and recovery time ~ 0.8 s. However, series combination lag in humidity sensing range of 80–100% RH. Solution for all range RH with linear response was further improved by introducing three humidity sensors in series by using PEDOT:PSS, Methyl Red, and Graphene Oxide^23^. This approach helps to achieve all range humidity responses between 0–100% RH. However, sensor lag in linearity with slow recovery response 3.5 s and fast response time 1 s. The MoSe_2_ and PVOH series sensor provide a linear response in all ranges with fast response and recovery time 0.6 s and 0.9 s, respectively^11^. In real-life conditions, humidity sensors are highly affected by environmental conditions like temperature, gases (N_2_, NH_3_, H_2_S, etc.), which become the main hurdle to achieve linear response for a longer time. This work provides a solution to solve sensor variation problems using linear combination techniques based on signal processing to achieve a linear response. The wider piecewise linear function in a range of 0–100% RH can be obtained by the sensor elements combination in rows and columns with fast response and recovery time 0.2 s and 0.4 s, respectively. This approach opens a gateway to design humidity sensors for the different operating conditions using the same material by adopting different combinations in sensing array with a signal processing technique.

**Table 1:** Comparison of Graphene and array based humidity sensors.

No.	Material type	Sensor type	Fabrication process	Range	Response time (s)	Recovery time (s)	Article reference
1	Graphene and ZnO nanocomposite	Impedance (2 parallel combination sensors)	Inkjet printing and spin coating	0–85% RH non-linear	1	2	^2^
2	Graphene and Methyl Red composite	Resistance and capacitance based sensor	Inkjet printing and spin coating	5–95% RH Towards linearity	0.25	0.35	^3^
3	MoS_2_/PEDOT:PSS	Impedance (2 series combination sensors)	SAW-EHDA deposition	0–80% RH Towards linearity	0.5	0.8	^13^
4	PEDOT:PSS/Methyl Red/Graphene Oxide	Impedance (3 series combination sensors)	Inkjet printing and spin coating	0–100% RH Non-linear	1	3.5	^23^
5	MoSe_2_/PVOH	Impedance (2 series combination sensors)	Screen printing and spin coating	0–100% RH Linear	0.6	0.9	^11^
**6**	**Methylene Blue and Graphene composite**	**Impedance (3 × 3 signal processed array; 9 sensors)**	**Inkjet printing and spin coating**	**0–100% RH highly linear**	**0.2**	**0.4**	**This work**

## Conclusion

As the concluding remarks, we presented all range inkjet printed linear and wide range humidity sensing 3 × 3 array with different interspaces of IDEs. The three different composite inks MB/BGF, MB/GF, and MB/GQD with different particle sizes were used for 3 × 3 array processed sensing. The impedance response of each sensor in the sensor array was analyzed at 1 kHz. The highly linear and wide-range response is achieved in a range of 0–100% RH with linear combination by using the signal processed technique. Every column and row with different electrode spacing or particle sizes, respectively, were physically connected in parallel, and the sensor arrays with the wide piecewise linear were demonstrated. The proposed sensor array shows fast response time and recovery time ~ 0.28 and 0.48, respectively. The proposed sensor array opens a new gateway for environment sensing systems, which can provide the related information with design parameters for all range linearity.

## Supplementary Information

Supplementary Information.
