# Supplementary material for: Wide range and highly linear signal processed systematic humidity sensor array using Methylene Blue and Graphene composite
Source: Sci Rep. 2021 Aug 17;11:16665. doi: 10.1038/s41598-021-95977-6 (PMC8371138; doi:10.1038/s41598-021-95977-6)
Supplement: Supplementary file 1 — Supplementary Information. [file 41598_2021_95977_MOESM1_ESM.docx]

Supplementary Information:

**Wide range and highly linear signal processed systematic humidity sensor array using Methylene Blue and Graphene composite**

Muhammad Umair Khan^a^, Gul Hassan^a,b^, Rayyan Ali Shaukat^a^, Qazi Muhammad Saqib^a^, Mahesh Y. Chougale^a^, Jungmin Kim^a^ and Jinho Bae^a*^

^a^Department of Ocean System Engineering, Jeju National University, 102 Jejudaehakro, Jeju 63243, Korea

^b^Centre for Advanced Electronics & Photovoltaic Engineering (CAEPE), International Islamic University, H-10, Islamabad 44000, Pakistan

^*^E-mail: baejh@jejunu.ac.kr

**Humidity sensing array fabrication method.**

**
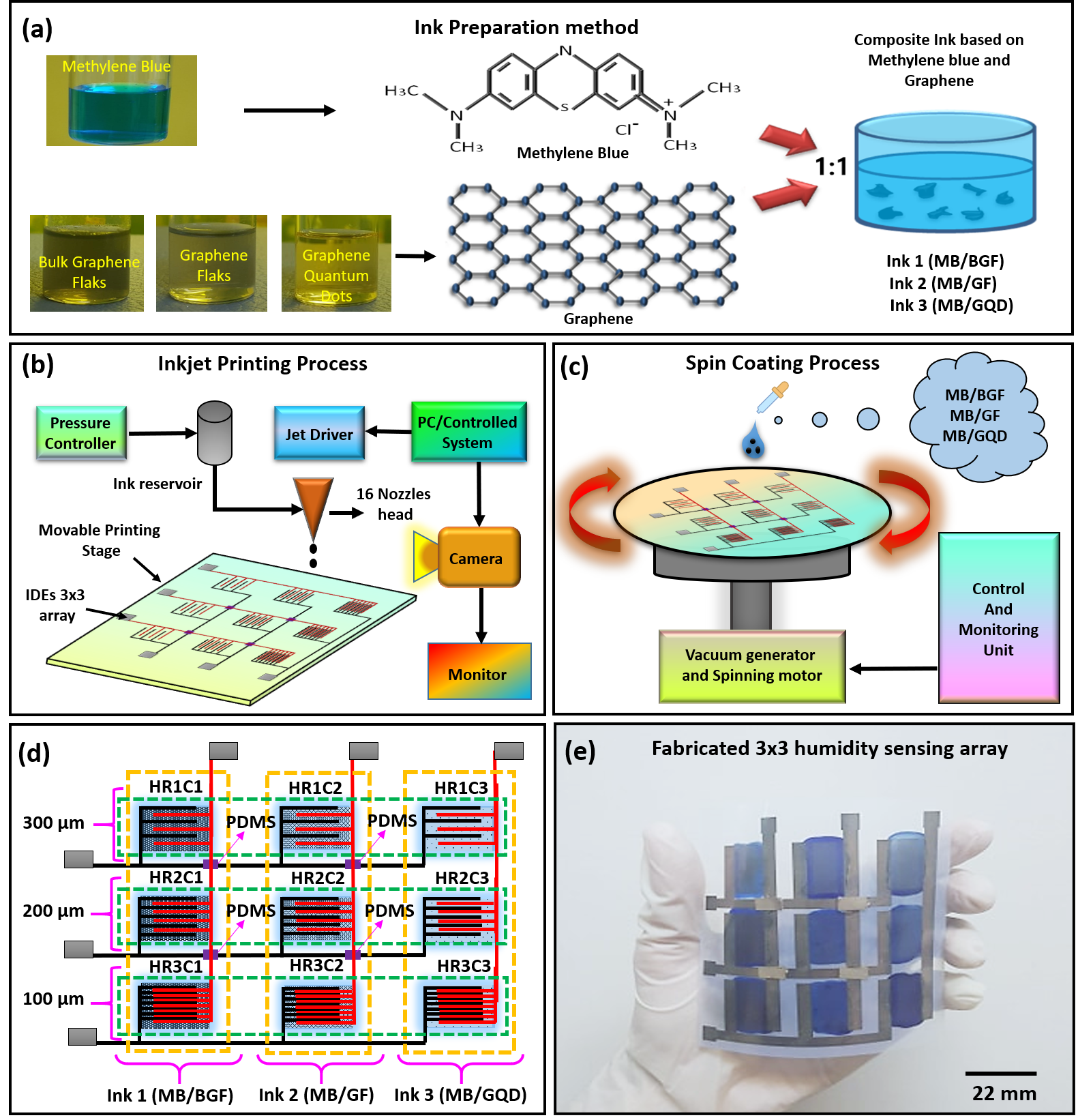
**

**Fig. S1. (**a) The ink preparation of Graphene different particles size with MB. (b) Fabrication IDEs 3×3 humidity sensor array using the DMP-3000 inkjet printer. (c) Fabrication of the sensing active layer using a spin coater. (d) schematic illustration of 3×3 humidity sensor array showing IDEs spacing and coated ink on each column. (e) Realized image of the fabricated sensor array.

**Equivalent model.** Each element in the circuit is presented in a complete sensor configuration. Here in Fig. S1 HR1C1 to HR3C3 presents sensor 1 to sensor 9 located in a 3×3 sensing array. Similarly, for all individual sensor circuits, R displays resistances, and C presents capacitances for sensors HR1C1 to HR3C3. Here, CPSE is the parasitic capacitance between the positive terminal and the substrate material added in series to the equivalent circuit. Upon energization through external circuitry, all sensor elements form parallel combinations within the sensor array, forming an equivalent circuit shown in Fig. S2a.

Thus, the sensing layer sheet resistances and capacitances get added up in parallel. The parallel combination reduces the array sensor sheet resistance and increases the terminal capacitance. The magnitude of the parallel sensing array is given by Eq. (1) where Req is the equivalent terminal resistance and Ceq is the terminal equivalent capacitance.

$$\left| Z_{1} \right|=\frac{1}{\sqrt{\frac{1}{{Req}^{2}}-\left( 2\pi fCeq \right)^{2}}} (1)$$

Magnitude of parasitic capacitance between the positive terminal and substrate material is given by Eq. (2).

$$\left| Z_{2} \right|=\frac{1}{\sqrt{\left( 2\pi fCPSE \right)^{2}}} (2)$$

Thus, both impedances add up in series giving the total impedance magnitude given in Eq. (3):

$\left| Z_{Total} \right|=\left| Z_{1} \right|+\left| Z_{2} \right|$ (3)

Upon energizing terminals, a potential difference is created between the IDEs as shown in a cross-sectional view in Fig. S2b. The electrodes at higher potential are displayed in red while the low potential electrodes are presented in blue as shown in Fig. S2c. Thus, an electric field is created between the electrodes originating from the higher potential surface and ending at low potential presented in 3D view in Fig. 2d. The arrows present the direction of the electric field is towards the ground electrode. This directional field aligns the H_3_O^+^ and OH^-^ ions in a polar molecular structure above the active layer. With the increase in adsorption of hydronium and hydroxyl ions the electric field intensity increases as shown in Fig. S2e.

**
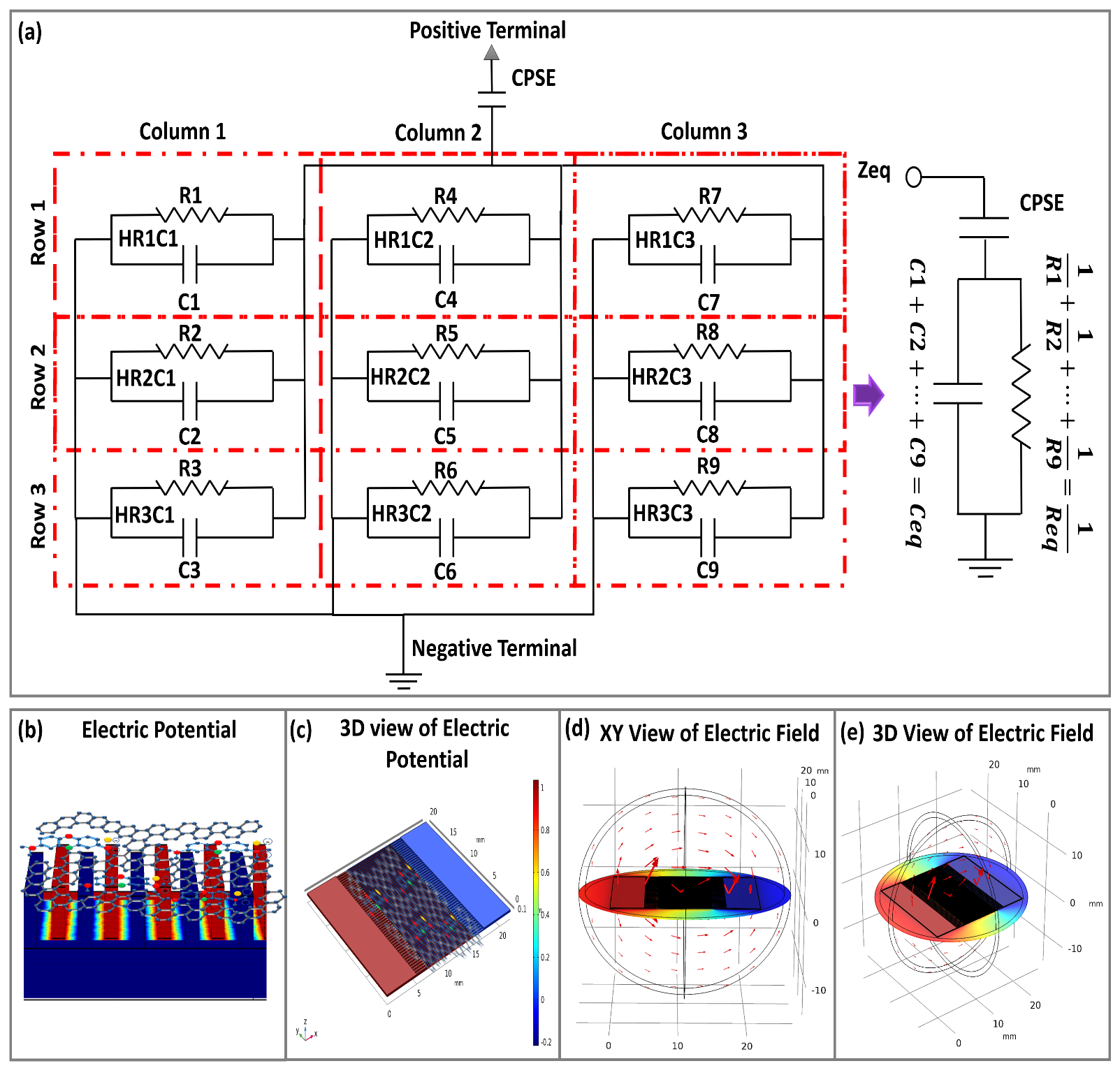
**

**Fig. S2. (a)** Equivalent model of 3×3 sensing array, (b) electric potential, (c) 3D view of electric potential, (d) XY view of electric field and, (e) 3D view of Electric field.

**IDEs surface morphology.** The surface morphology of IDEs is shown in Fig. S3a with a magnification level of 100 μm, which ensures that electrodes are properly fabricated with inkjet DMP-3000 printing technology. The zoomed image of Ag is shown in Fig. S3b with a magnification level of 1 μm, which ensures that Ag nanoparticles are properly connected and properly centered at 100 ^o^C. The EDS spot profile is performed to confirm the nature of IDEs as shown in Fig. S3c. The Ag peak can be observed at 3.01 keV and the PET peak is observed at 0.12 keV. The EDS layered image of IDEs is and PET substrate is analyzed at a magnification level of 1 μm as shown in Fig. S3d, with Ag L Series as shown in Fig. S3e and C K series as shown in Fig. S3f. The 2D nano-profile of IDEs is performed to confirm the roughness (Ra). The roughness of Ag electrodes is Ra ~ 2.61 μm as shown in Fig. S3g. The 3D nano-profile is performed to confirm the height profile of IDEs. The height profile of IDEs is 4.08 μm as shown in Fig. S3h. The height profile of IDEs also confirms the height profile of IDEs as shown in Fig. S3i.

**
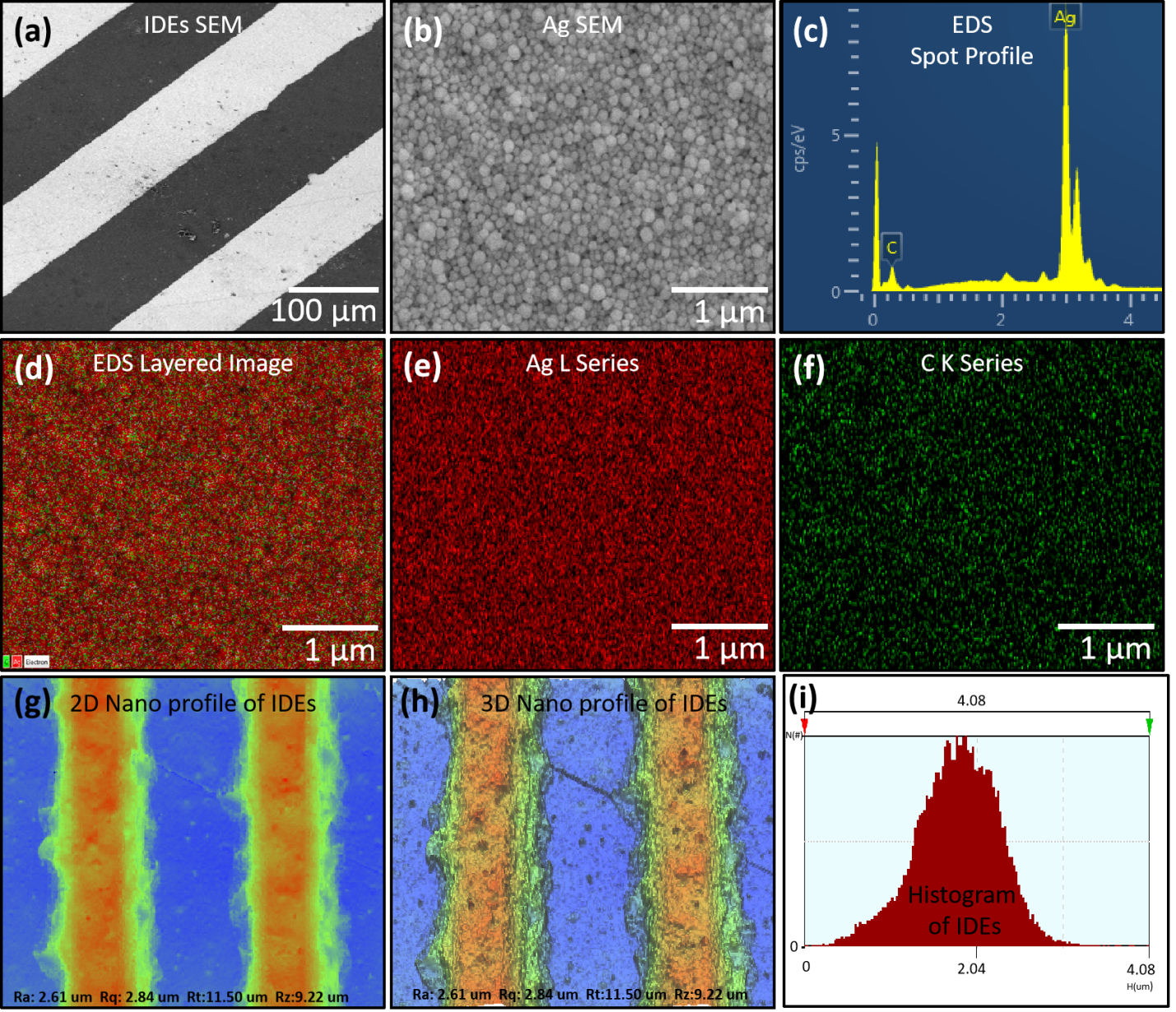
**

**Fig. S3.** (a) Surface morphology of IDEs showing proper fabrication using an inkjet printer. (b) SEM image of Ag at 1 μm showing electrodes are properly cured and particles are connected with each other. (c) EDS spot profile of IDEs and PET substrate. (d) EDS layered image of IDEs showing (e) Ag L series and (f) PET substrate showing C K series. Nano-profile of IDEs is showing (g) surface roughness and (h) height profile. (i) Histogram of IDEs.

**MB surface morphology.** The surface profile of MB is shown in Fig. S4a with a magnification is 1 μm. The surface morphology of MB is highly uniform, and the film is uniformly fabricated using a spin coater without any cracks and pores. The Element composition of MB is confirmed using the EDS spot profiler, which confirms the C peak at 0.12 keV and N peak at 0.21 keV and O k peak at 0.5 keV and S k peak at 2.14 keV and Cl k 2.31 keV as shown in Fig. S4b. The EDS mapping of MB is shown in Fig. S4c with a magnification level of 1 μm, which confirm the presence of C K series as shown in Fig. 4d, O K series in Fig. S4e, S K series as shown in Fig. S4f and Cl K series in Fig. S4g and N K series shown in Fig. S4h. Fig. S4i shows the Raman spectra of MB in which two characteristic peaks at 1624 cm^-1^ are attributed to the C-C ring stretching and 1395 cm^-1^ are attributed to C-N symmetrical stretching. In-plane ring deformation mode of C-H is observed at 1302 cm^−1^, while in-plane ring bending mode of C-H is at 1154 cm^−1^ and 770 cm^−1^. The C-N-C skeletal deformation mode peaks are observed at 501 cm^−1^ and 448 cm^−1^ [1].

**
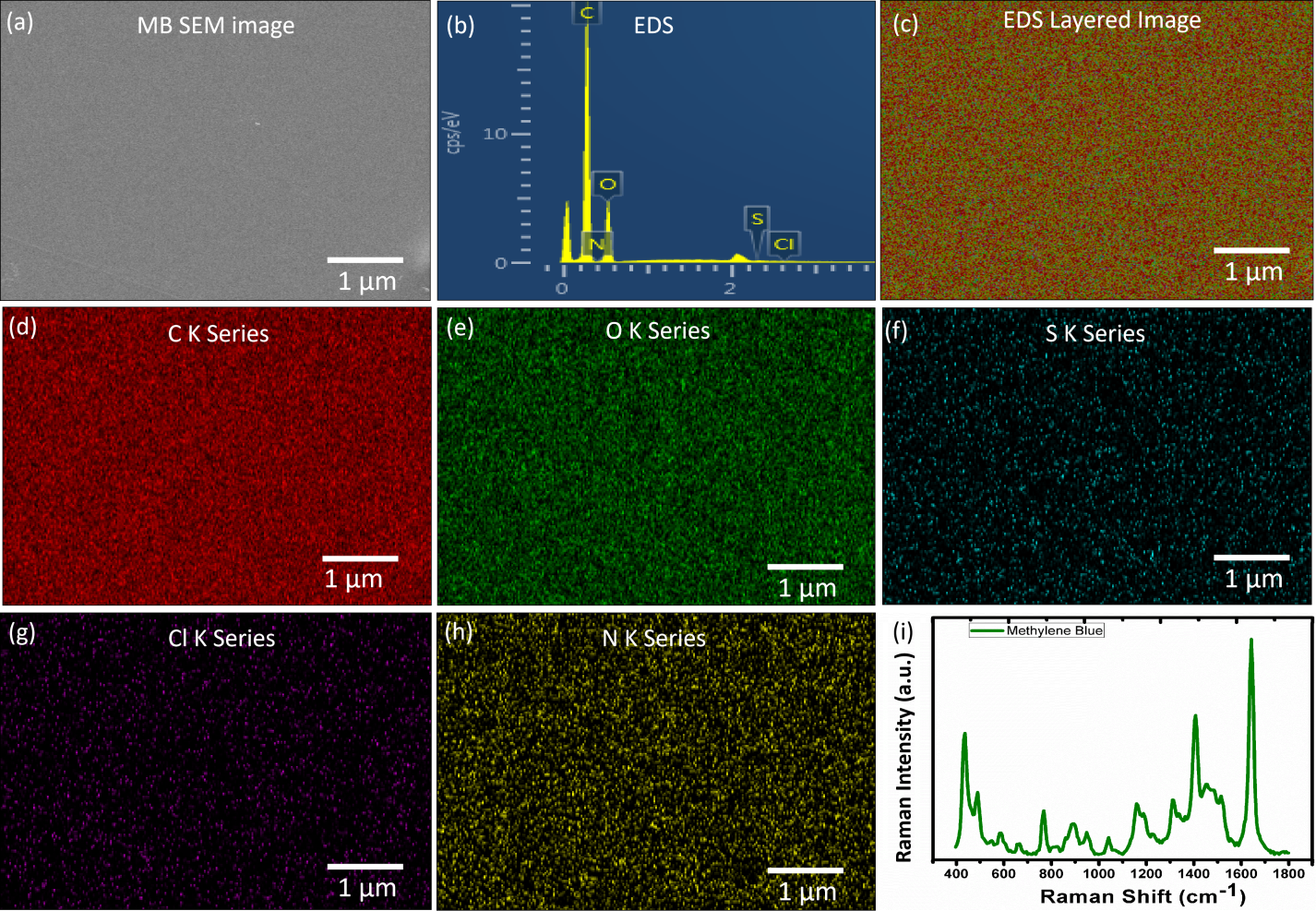
**

**Fig. S4.** (a) The surface morphology of MB at 1 μm. (b) EDS spot profile of MB. (c) The EDS layered image showing (d) C K series, (e) O K series, (f) S K series, (g) Cl K series, and (h) N K series at 1 μm. (i) Raman shift of MB.

**Graphene different particle size surface morphology.** The element composition of Graphene is confirmed using the EDS spot profile as shown in Fig. S5a, which confirms the C peak at 0.13 keV with 100 wt%. The EDS layered image of Graphene is shown in Fig. S5b with a magnification level of 1 um showing the C K series. The surface morphology of bulk Graphene flakes is shown in Fig. S5c, with a magnification level of 1 μm, which clearly shows that Graphene flasks are present in bulk form and flacks are overlapping each other. The surface morphology of processed Graphene flakes is shown in Fig. S5d, which confirms that flakes are processed and particle size can be observed clearly, and flacks overlapping also decreased. The Surface morphology of GQD is shown in Fig. S5e with a magnification level of 1 μm, which confirms that the film is uniform. The main reason for the film uniformity using spin coater is the size of GQD which is 5 nm. The Graphene is further evaluated for chemical bonding using the Raman spectrum as shown in Fig. S5f. The D peak is observed at 1355 cm^-1^ and the G peak is observed at 1596 cm^-1^ [2].

**
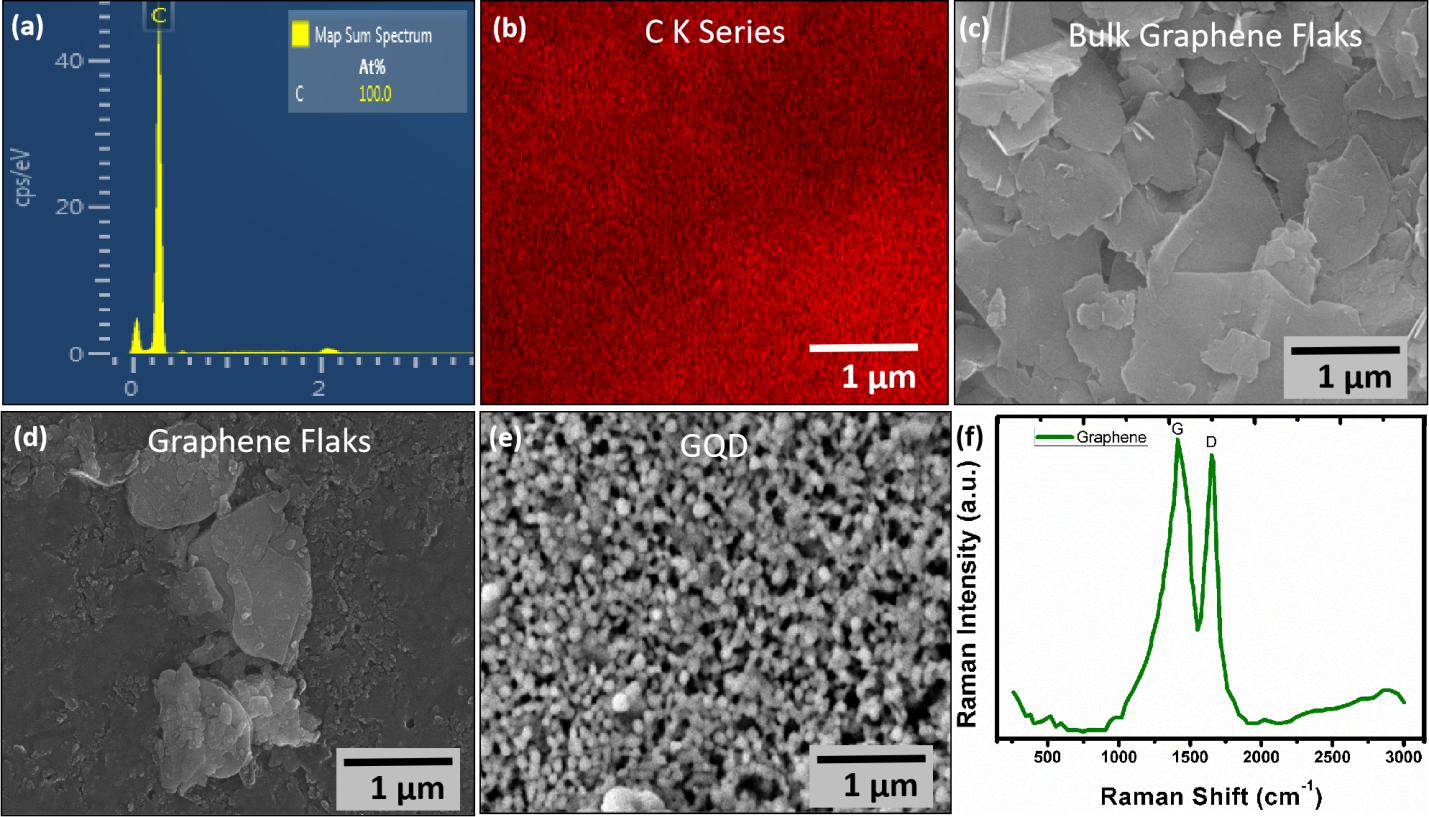
**

**Fig. S5.** (a) The EDS spot profile of Graphene. (b) EDS layered image of Graphene showing C K series at 1 μm. Surface morphology of (c) Bulk Graphene flakes at 10 μm, (d) Graphene flakes at 1 μm, and (e) Graphene quantum dots at 500 nm. (f) Raman shift of Graphene.

**Humidity analysis.** The impedance response of the sensing array towards change in relative humidity was recorded in a homemade environmental-controlled chamber. The volume of gas at the chamber inlet was controlled through an electronic mass flow controller (MFC) from 0% RH to 100% RH. The humidifier is used for humidification from 0% to 100% RH and the air compressor is used for dehumidification from 100% to 10% RH and for further dehumidification dry nitrogen (N_2_) is used from 10% to 0% RH. After there is slowly reaching an increment of impedance response ~ 1% RH, the system was stabilized for 10 s and then move towards the next data logging point. The KEYSIGHT U1700C LCR meter with 0.6 Vrms AC output was used to measure the change in impedance level of each element in a sensing array, Arduino board is used for data acquisition from the reference sensor HTU21D with a resolution of 0.04% RH. The data of the reference humidity measured from HTU21D and the 3×3 sensor array responses were continuously logged through the USB communication interface. The sensor array data were logged with built-in software of KEYSIGHT U1700C LCR meter and the reference sensor data was logged performing with cool term software For graph plotting of the measured data, the origin pro 8.0 was used. The experiment was performed at 25 °C (room temperature), and its schematic of the characterization setup is composed as shown in Fig. S6.

**
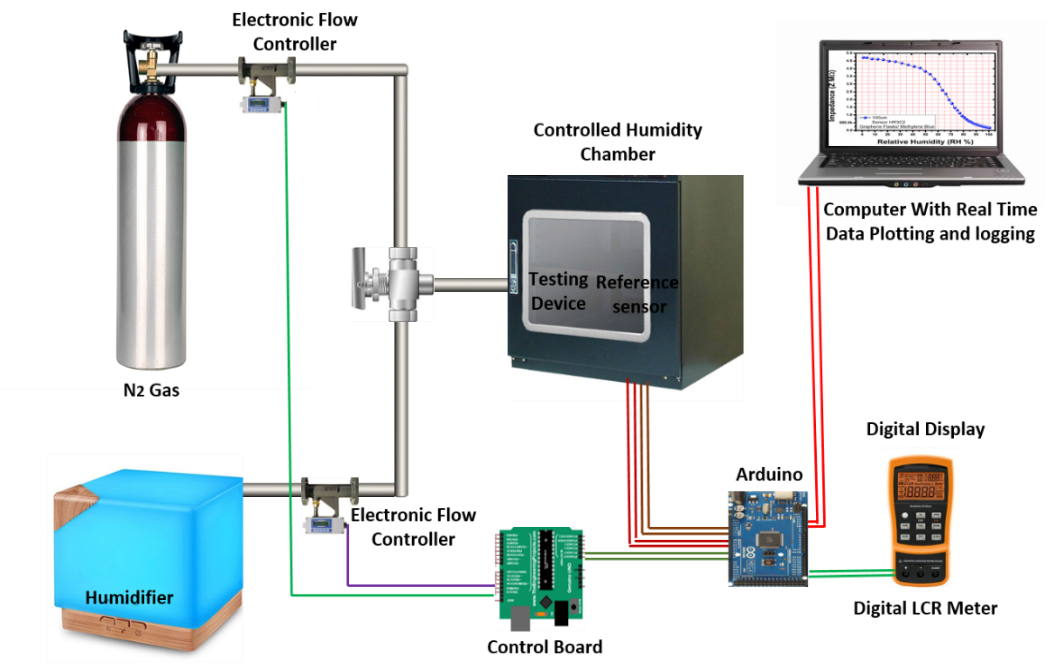
**

**Fig. S6.** Schematic of the humidity sensing setup.

**Sensor mathematical modeling and array Processing.** In many kinds of literature [3], an impedance mathematical model of IDEs humidity sensor was proposed as

$$Z=R+\frac{1}{j2\pi fC} (4)$$

where, $j=\sqrt{-1}$, *f* is the frequency, *R* is the resistance of wires, *C* is the capacitance. Hence, this paper proves the mathematical model of IDEs. To prove this model, the resistance, capacitance, and impedance were measured from bulk Graphene flakes composite (MB/BGF) sensor on 100 μm interspacing electrode as shown in Fig. S7.


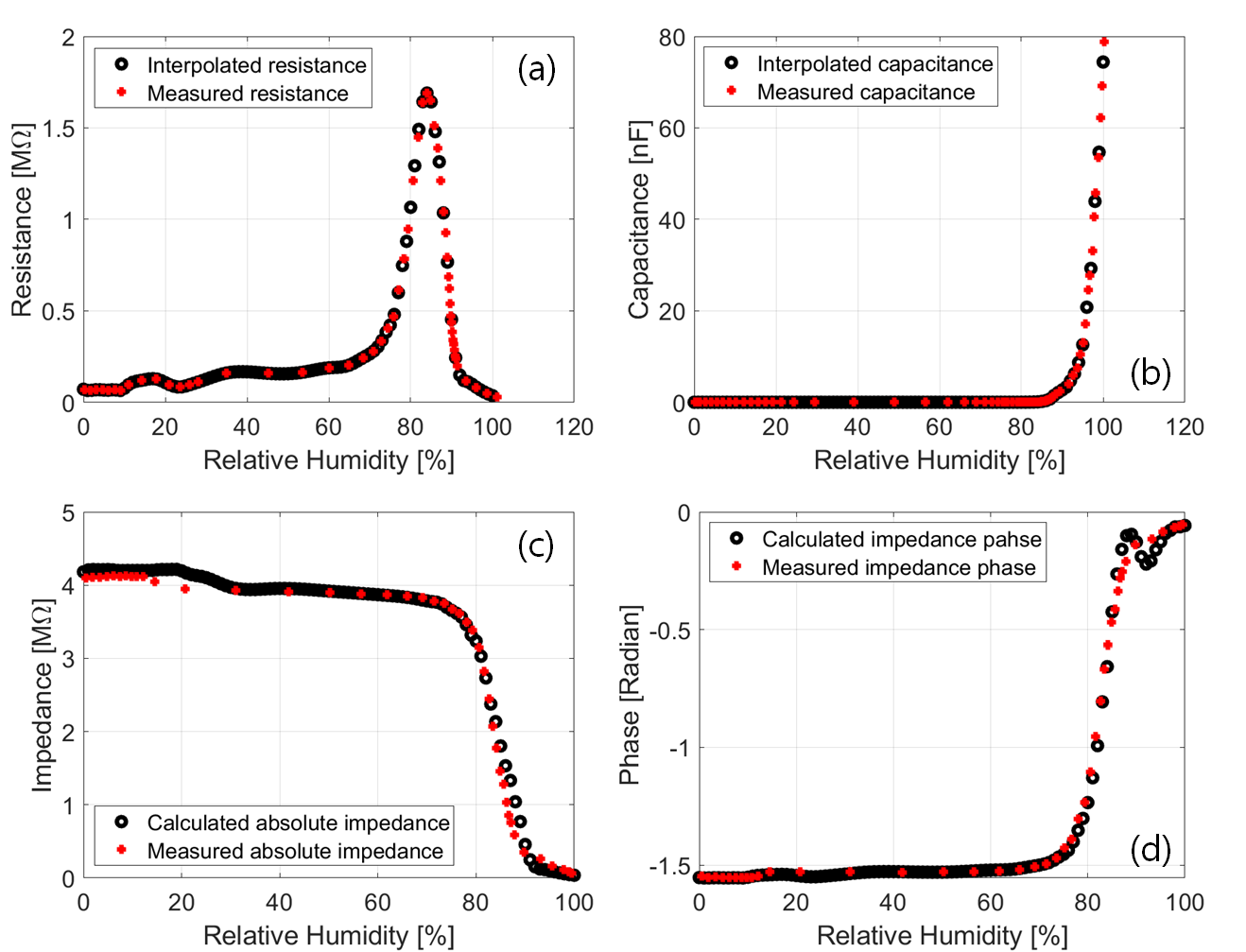


**Fig. S7**. (a) The measured resistance and the interpolated resistance from it by spline interpolation. (b) The measured capacitance and the interpolated capacitance from it by spline interpolation. (c) The calculated absolute values of impedance from the mathematical model and the measured absolute impedance. (d) The calculated phase values of impedance from the mathematical model and the measured phase values.

Here, resistance and capacitance are measured as red crosses shown in Fig. S7a and S7b, respectively. There have not same sampled intervals because our LCR meter cannot measure simultaneously, so the data are matched the same and uniform interval as 1% RH by using spline interpolation method [4] as black circles shown in Fig. S7a and S7b. Utilizing these data, the impedance is calculated from Eq. (4), and the absolute impedance ($\left| Z \right|$) and their phase information are obtained as black circles shown in Fig. S7c and S7d. All results for supplementary information were calculated on Matlab R2019a. These are compared with the randomly measured absolute impedance (red crosses in Fig. S7c) and phase information (red crosses in Fig. S7d) as shown in Fig. S7c and S7d. From these comparisons, we can see that this mathematical model of IDEs humidity sensor is valuable because there are matched quite well. Here, due to an effect for this model of inductance values is relatively very small, these values can be approximately removed.

Here, we measured data from the fabricated 3×3 humidity array. Total measured 100 data are obtained by 1% uniform sampling interval in between 0% to 100% relative humidity (RH) at 1 kHz. Here, all sampled data are not uniform along with RH because keeping the same and uniform RH interval is difficult to detect the impedance, so the uniform data are obtained by the spline interpolation method [5] from the randomly measured data. From these obtained data, we can assume that each sample data is function as $\left\{ f_{\mathrm{mn}}(\mathrm{RH}_{i}) \right\}$, where m = {1, 2, 3}, n = {1, 2, 3}, and RH_i_ is a relative humidity at *i*th sample point ($i=1, 2, \cdots, 100$). Using these measured data, we can make an as following equation.

$f_{m}\left( \mathrm{RH}_{i} \right)=a_{11} f_{11}\left( \mathrm{RH}_{i} \right)+a_{12} f_{12}\left( \mathrm{RH}_{i} \right)+\cdots+a_{32} f_{32}\left( \mathrm{RH}_{i} \right)+a_{33} f_{33}\left( \mathrm{RH}_{i} \right)\approx Z_{i}$ (5)

From above (5), we can manipulate a matrix formulation as (6).

$\left[ \begin{matrix} \begin{matrix} f_{11}(\mathrm{RH}_{1}) \\ f_{11}(\mathrm{RH}_{2}) \\ \begin{matrix} \vdots\\ f_{11}(\mathrm{RH}_{N}) \end{matrix} \end{matrix} & \begin{matrix} f_{12}(\mathrm{RH}_{1}) \\ f_{12}(\mathrm{RH}_{2}) \\ \begin{matrix} \vdots\\ f_{12}(\mathrm{RH}_{N}) \end{matrix} \end{matrix} & \begin{matrix} \begin{matrix} \cdots\\ \cdots\\ \begin{matrix} \ddots\\ \cdots\end{matrix} \end{matrix} & \begin{matrix} f_{33}(\mathrm{RH}_{1}) \\ f_{33}(\mathrm{RH}_{2}) \\ \begin{matrix} \vdots\\ f_{33}(\mathrm{RH}_{N}) \end{matrix} \end{matrix} \end{matrix} \end{matrix} \right]\left[ \begin{matrix} a_{11} \\ a_{12} \\ \begin{matrix} \vdots\\ a_{33} \end{matrix} \end{matrix} \right]=\left[ \begin{matrix} Z_{1} \\ Z_{2} \\ \begin{matrix} \vdots\\ Z_{N} \end{matrix} \end{matrix} \right]$ (6)

where N = 100 and the ideal all range linear values, $\left\{ Z_{i}=-4.9536\times{10}^{4}{RH}_{i}+5.4195\times{10}^{6} \right\}_{i=1}^{N}$ were obtained using the average of the 9 impedances from all sensors at 0% and the average impedance at 100% from the measured datum of the 9 samples in Fig. S8. Let Eq. (6) is $A\underline{a}=\underline{Z}$, then the best estimate weight values, $\underline{\hat{a}}$ can be calculated applying the least square solution [3] as $\underline{\hat{a}}={(A^{T}A)}^{-1}A^{T}\underline{Z}$, where, T is a transpose.

The estimated weight values were calculated as $\underline{\hat{a}}=\{\hat{a}_{11}, \hat{a}_{12}, \hat{a}_{13}, \hat{a}_{21}, \hat{a}_{22}, \hat{a}_{23}, \hat{a}_{31}, \hat{a}_{32}, \hat{a}_{33}\}$ = {-1.395919139453048, 1.509000728240909, 12.98669965832261, 0.521398705626367, 1.16948647255811, -2.648766910355043, -12.53225673113707, -0.002212571475620711, 2.067765966401583e} as shown in Fig. 3 in main manuscript.


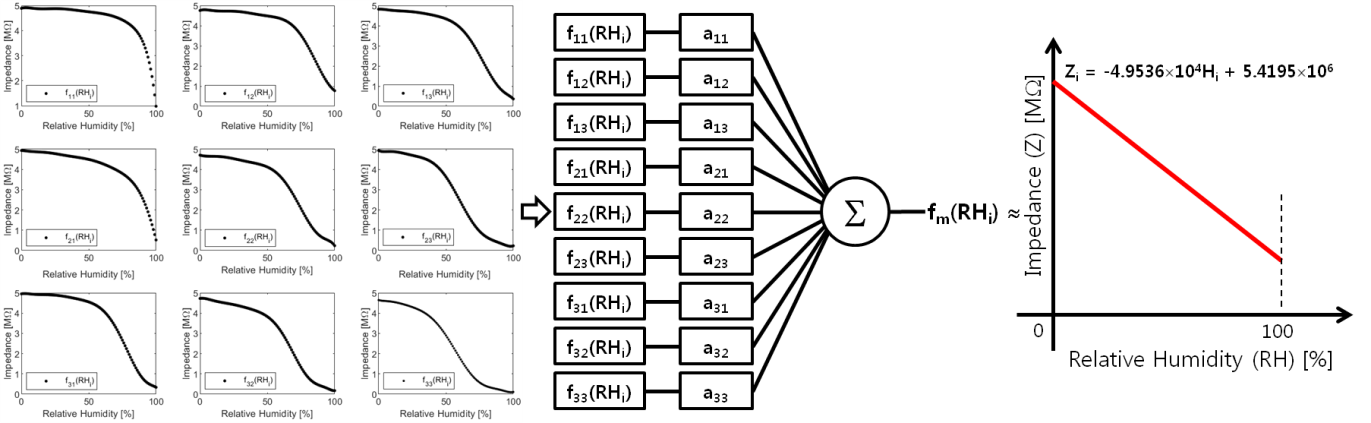


**Fig. S8**. Schematic diagram to calculate weight values.

**References**

[1] G.-N. Xiao and S.-Q. Man, "Surface-enhanced Raman scattering of Methylene Blue adsorbed on cap-shaped silver nanoparticles," Chemical Physics Letters, vol. 447, pp. 305-309, 2007/10/25/ 2007.

[2] F. T. Johra, J.-W. Lee, and W.-G. Jung, "Facile and safe Graphene preparation on solution based platform," Journal of Industrial and Engineering Chemistry, vol. 20, pp. 2883-2887, 2014/09/25/ 2014.

[3] S. Ali, A. Hassan, G. Hassan, J. Bae, C.H. Lee, All-printed humidity sensor based on Graphene/methyl-red composite with high sensitivity, Carbon 105 (2016) 23-32.

[4] J. Stoer, R. Bulirsh, Introduction to Numerical Anaysis, Springer-Verlag, NY, 1980.

[5] G. Strang, Linear Algebra and Its Applications, 4th ed., Thomson Brook/Cole, CA, 2006.
